# Knockdown of long noncoding RNA AL161431.1 inhibits malignant progression of cholangiocarcinoma

**DOI:** 10.18632/aging.205898

**Published:** 2024-08-01

**Authors:** Zhoulan Bai, Na Tian, Zhe Ding, Qing Lu, Yuchen Wang, Shangting Du, Yongfeng Hui

**Affiliations:** 1 Department of Radiation Oncology, General Hospital of Ningxia Medical University; Cancer Institute, Ningxia Medical University, Yinchuan 750004, Ningxia, PR China; 2 Department of Cardiology, Ningxia Medical University, Yinchuan 750004, Ningxia, PR China; 3 Department of Hepatobiliary Surgery, General Hospital of Ningxia Medical University, Yinchuan 750004, Ningxia, PR China

**Keywords:** cholangiocarcinoma, lncRNA, epithelial-mesenchymal transition, immune

## Abstract

Background: Cholangiocarcinoma (CCA) is one of the most deadly cancers in the world. It usually has a bad prognosis and is challenging to identify in its early stages. Long noncoding RNAs (lncRNAs) have been shown in an increasing number of studies to be important in the control of signaling pathways, cell behaviors, and epigenetic modification that contribute to the growth of tumors. The purpose of this work was to examine the relationship between CCA and lncRNA AL161431.1.

Methods: Using TCGA clinical survival data, we evaluated the association between AL161431.1 expression and patient prognosis. Using the program cluster Profiler R, enrichment analysis was performed. Additionally, the association between immune cell infiltration and AL161431.1 expression was evaluated by a review of the TCGA database. Next, to ascertain if AL161431.1 influences tumor growth, migration, and invasion in CCA, functional *in vitro* assays were conducted. Quantitative real-time polymerase chain reaction (qPCR) was employed to gauge AL161431.1 expression levels in CCA cells. Western blot was used to measure protein levels.

Results: In CCA, AL161431.1 was extremely expressed. The patients in the high-risk group had a significantly poorer overall survival (OS) than the patients in the low-risk group. A more thorough look at the TCGA data showed a relationship between high expression levels of AL161431.1 and increased infiltration of T cells, T helper cells, and NK CD56dim cells. Furthermore, AL161431.1 knockdown in CCA cells impeded invasion, migration, and proliferation and also lowered the expression of phosphorylated Smad2/Smad3 to restrain the TGFβ/SMAD signaling pathway.

Conclusions: Our results indicate that the lncRNA AL161431.1 activates the TGFβ/SMAD signaling pathway to enhance CCA development and metastasis. AL161431.1 could be a novel target for cholangiocarcinoma treatment or a diagnostic marker.

## INTRODUCTION

Cholangiocarcinoma (CCA) is a highly lethal kind of cancer worldwide. [1]. It often has a bad prognosis and is challenging to diagnose early [2]. The prevalence of CCA has increased worldwide in recent years and is higher in Asian countries than in Western countries, despite accounting for less than 15% of liver primary cancer [3]. Surgical resection remains the primary line of treatment for CCA because of a lack of sensitivity to radiation and chemotherapy. Despite this, the five-year survival rate for CCA is merely 10%, indicating a high probability of post-operative recurrence [4]. Therefore, comprehending the molecular pathways behind the evolution of CCA is essential.

LNCRNAs are a kind of longer than 200 nt regulatory RNAs with restricted ability to code for proteins. LNCRNAs interact with DNAs, miRNAs, or proteins to regulate a variety of physiological and pathological cellular processes at the transcriptional and post-transcriptional stages [5–7]. In CCA, an increasing amount of research showed that lncRNAs have been linked to epigenetic modification, signaling pathway control, and cell behaviors that promote tumor growth [8]. Even though there are many LNCRNAs in the human genome, only a tiny portion of them have been functionally investigated. This presents a chance to find new targets for therapeutic intervention in CCA patients. In our study, we identified a CCA-associated up-regulated lncRNA AL161431.1 in cancerous bile and tissues via lncRNA microarray, which is an intergenic lncRNA located on chr13:109921982-109926186. Through the use of CCA cell lines in biological tests and bioinformatics analysis, the involvement of AL161431.1 in the etiology and prognosis of CCA was investigated. The results indicated that AL161431.1 could promote CCA cells proliferation, migration and invasion. Furthermore, AL161431.1 increased the expression levels of phosphorylated smad2/smad3 to activate the TGFβ/SMAD signaling pathway in CCA cells.

## MATERIALS AND METHODS

### Bioinformatic analysis

From TCGA, transcriptome data, including mRNA and lncRNA, as well as clinical information about cholangiocarcinoma patients, were obtained. The OS was defined as the period of time from the patients’ CCA diagnosis until their death or the study’s completion. Kaplan-Meier analyses involving log-rank tests were used to measure survival. The edgeR technique was utilized to analyze differential mRNA abundance and derive the log2FC values for every gene. Genes with read counts greater than 2 were included in the final quantitative and statistical analysis. Using Cytoscape, the protein interaction network of the aforementioned differential genes was built, with a confidence cutoff of 0.4. Gene enrichment analysis was performed with GSEA. For GSVA analysis, the hall-markers gene set was retrieved. We further retrieved 24 immune cell gene sets from a 2013 Immunity publication titled “Synamotemporal Dynamics of Intra-tumoral Immune Cells Reveal the Immune Landscape in Human Cancer” to look into the possible relationship between immunity and AL161431.1.

### Cell culture and cell transfection

ScienCell Research Laboratories (Beijing, China) provided the human normal bile vessel cell line HIBEpic, and the CCA cell lines RBE, KMBC, and QBC-939, were acquired from the Shanghai Cell Bank of the Chinese Academy of Sciences. CCA cell lines were cultured at 37°C in a 1640 medium (Gibco, USA) mixed with 10% FBS and 100 U/ml of penicillin and streptomycin. Following a 20-minute room temperature incubation period, 5 μl of lipofectamine 3000 (Cat#L3000001, Invitrogen, Shanghai, China) was mixed with 2 μg of siRNAs targeting AL161431.1 in 125 μl of RMPI 1640 medium. The cells were extracted for the ensuing tests after a 48-hour transfection period. Table 1 comprised the siRNA sequences.

**Table 1:** Sequences of the FISH probes and siRNA used in this study.

AL161431.1(FISH)	Cy3-TGGGTCATGGCAATGTCAGTGAAGGT
18s	FAM-CTGCCTTCCTTGGATGTGGTAGCCGTTTC
si- AL161431.1-1	CCTCCAACACTTTGACTAT
si- AL161431.1-2	GCCTGTTCTTCAGAAGGAA
si- AL161431.1-3	CAACCTCCACCTTGCTTAA

### Quantitative real-time PCR (qPCR) assay

Using RNAiso Plus (Takara, Dalian, China), total RNA was extracted from cells in accordance with the manufacturer’s recommendations. An ultra-microUV spectrophotometer from Thermo Fisher Scientific (Waltham, MA, USA) was used to measure the concentration and purity of total RNA. Utilizing the PrimeScript RT reagent Kit (Takara, Dalian, China), reverse transcription was performed. The qPCR was performed using the Hieff qPCR SYBR Green Master Mix kit (Yeasen, Shanghai, China) The findings were analyzed using the 2^−ΔΔCt^ technique. GAPDH served as our internal benchmark. Table 2 displays relevant primer sequences for quantitative PCR.

**Table 2:** Sequences of the qRT-PCR primers used in this study.

**Gene name**	**Forward primer (5′–3′)**	**Reverse primer (5′–3′)**
LINC00973	CTCTTGGGAGCATGTGGACAGTTG	CCTCAGACCAGGAAGCCTTCAATTC
AL161431.1	CAGACAGCACAGCCACTTCACC	CATGGAGAGACTGGAGCCGAGAG
AC006504.8	TGAGCAGAAGCCTCACGTAATTAG	CAGGTGTTTGAACAACTGAACTGAA
LINC01336	GCCCTTCCCAGAGACCAGTT	CTCTTAGGTTTTAGAGCTTGGGGG
AL365356.5	AGTGGGTGAAGTCCTGGTTC	TGCAGACCACCATAAGGGAT
AC010735.2	GAAGGGTTGAGGGTACCAAGG	TTTTCTCACTGCCCCAGGAAA
AC002384.1	CTCCCCTTTTTGCCGGGAT	TGCAGACCACCATAAGGGATT
U6	ATTGGAACGATACAGAGAAGATT	GGAACGCTTCACGAATTTG
GAPDH	AACGGATTTGGTCGTATTGG	TTGATTTTGGAGGGATCTCG

### Western blot analysis

Following the extraction using RIPA and PMSF, the concentration of the total cellular protein was determined using the BCA Protein Assay Kit (Solarbio, Beijing, China). Equal amounts of protein were loaded, separated, and then transferred to a PVDF membrane on a 10% SDS-PAGE gel. After being blocked with 5% BSA in TBST, PVDF was rinsed and incubated at 4°C overnight with primary antibodies from Proteintech, Wuhan, China (smad2, smad3, smad4, GAPDH), and Cell Signaling Technology, Danvers, MA, USA (phospho-SMAD2, phospho-SMAD3). The primary antibodies were then hybridized with a secondary antibody (1:10000) at 37°C for two hours. Ultimately, Quantity One software and ECL reagents were used to visualize the immunoreactions.

### Cell counting kit-8 assay

To quantify the rate of cell growth, the Cell Counting kit-8 from Dojindo Laboratories, Kumamoto, Japan was used. In all, 96-well plates were planted with 3000 treated CCA cells. Subsequently, 10 ul of CCK-8 reagent was diluted with 100 ul of medium and added to each well at the designated times (24, 48, 72, and 96 hours). After that, the cells were cultured for two hours at 37°C. A microplate reader (BioTek Instruments, Winooski, VT, USA) was used to calculate the optional density (OD) at 450 nm.

### 5-Ethynyl-20-deoxyuridine (EdU) incorporation assay

Treated cells (2 × 10^4^/well) were cultivated for 24 hours on 96-well plates. After being incubated for two hours at 37°C with fifty milligrams of EdU, the cells were fixed with 4% paraformaldehyde for twenty minutes. After that, the treated cells were given 100 μL of Click reaction cocktail, permeabilized using 1% Triton X-100, and stained with Hoechst 33342. Using an Olympus fluorescent microscope (Japan), immunostaining was imaged and quantitatively assessed.

### Colony formation assay

A density of 1000 transfected cells per well was used to inoculate 6-well plates, and the plates were cultured for two weeks at 37°C. Following a 30-minute fixation with 4% paraformaldehyde, the cells were stained for an additional 30-minute period using 1% crystal violet. The colonies were then numbered and photographed.

### Wound healing assay

After the cells attained 90–100% confluence in six-well plates, the wound field was scraped using a 200 μl pipette tip. Using high-power field photography, representative pictures of cell migration were captured 0 and 24 hours following damage.

### Migration and invasion assays

After the cells attained 90–100% confluence in six-well plates, 200 μl of serum-free medium with transfected CCA cells was added into the top chambers for Trans-well studies. The matrigel mix (BD Biosciences, Franklin Lakes, NJ, USA) was used for invasion testing. 600 μL of complete media containing 20% FBS was added to the bottom chambers as a nutritional attractant (Corning, NY, USA). 4 × 10^4^ CCA-modified cells were planted into the top chambers to perform migration and invasion assays. After being fixed for 24 or 36 hours in 4% paraformaldehyde (Beyotime, Shanghai, China), the cells on the compartment were stained with crystal violet (Solarbio, Beijing, China). An optical microscope was used to count and take pictures of them (Olympus, Tokyo, Japan).

### Nuclear and cytoplasmic extraction and fluorescence *in situ* hybridization (FISH)

Utilizing the PARISTM kit (Invitrogen, Thermo Fisher Scientific, Waltham, MA, USA) in accordance with the manufacturer’s instructions, the nuclear and cytoplasmic fractions of RNA were extracted. After then, qPCR was used to examine the RNAs in order to determine AL161431.1 expression. To identify the location of AL161431.1 in CCA cells, a FISH experiment was used. To summarize, cell climbing sections were prehybridized at 37°C for 30 minutes as per the manufacturer’s recommendations. Afterwards, 2.5 μL 20 μM specific Cy3-labelled AL161431.1 probes and FAM-labelled 18s probes were hybridized (GenePharma, Shanghai, China). The probe sequences were shown in Table 1. Slides were photographed using confocal laser scanning microscopy (Zeiss, Jena, Germany).

### Availability of data and material

The datasets used and/or analyzed during the current study are available from the corresponding author on reasonable request.

## RESULTS

### LncRNA AL161431.1 expression in CCA

**Figure 1:**
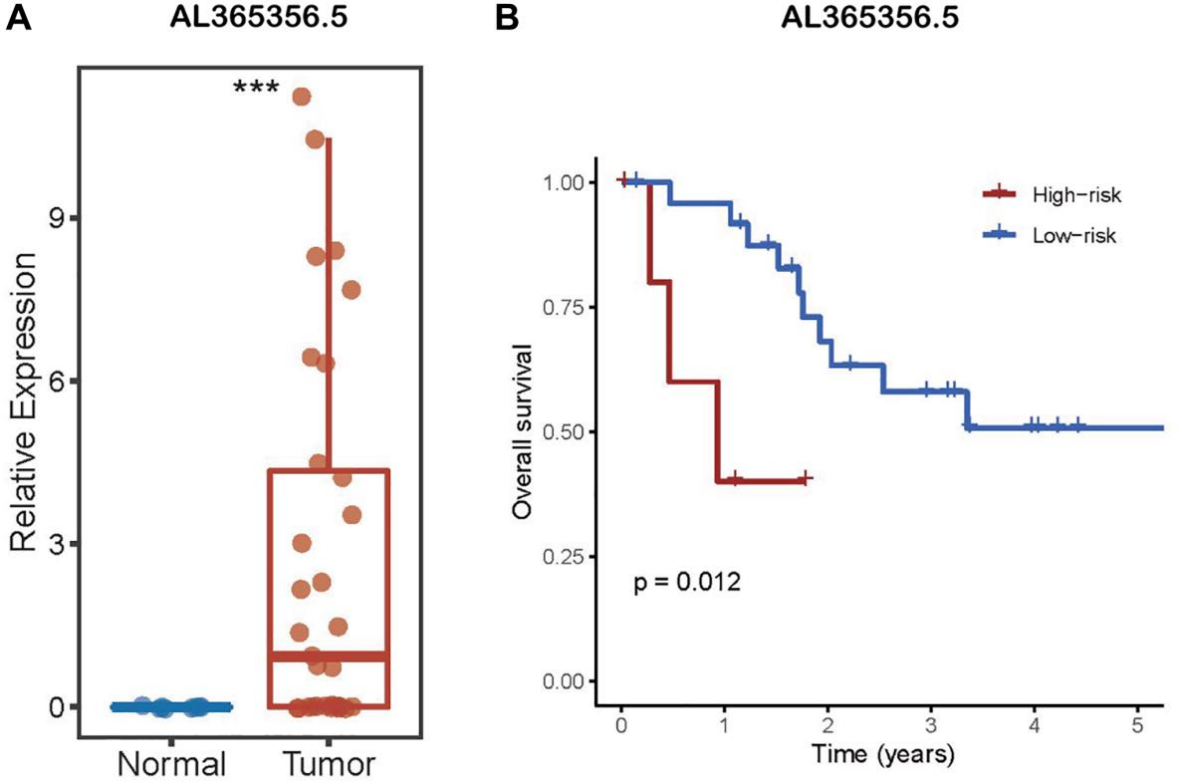
**LncRNA AL161431.1 expression in CCA.** (**A**) AL161431.1 was significantly up-regulated in CCA according to TCGA. (**B**) Kaplan–Meier analysis of OS in patients with CCA according to lncRNA AL161431.1 expression. ^ns^*p* ≥ 0.05, ^***^p < 0.001.

To conduct research to figure out whether AL161431.1 differs in CCA from the surrounding normal tissues, the CHOL transcriptome data from TCGA were downloaded, 39 tumor samples and 8 normal samples were ultimately retrieved after eliminating one sample that had undergone preoperative adjuvant chemoradiotherapy and one sample that came from non-bile duct tissue. As depicted in the Figure 1A, the expression of AL161431.1 was shown to be considerably greater in tumor samples compared to surrounding non-tumor tissue.

According to the aforementioned findings, lncRNA AL161431.1 may have high research value. As a result, TCGA provided the survival data for the CHOL samples, which were then classified into high- and low-risk groups based on the expression level of AL161431.1. The Kaplan-Meier curve was used for survival analysis. The results showed a favorable correlation between AL161431.1 expression level and the prognosis for CCA. (Figure 1B). Specifically, individuals with CCA who had higher expression levels of AL161431.1 had shorter OS.

### Prediction of the functions of LncRNA AL161431.1 in CCA

**Figure 2:**
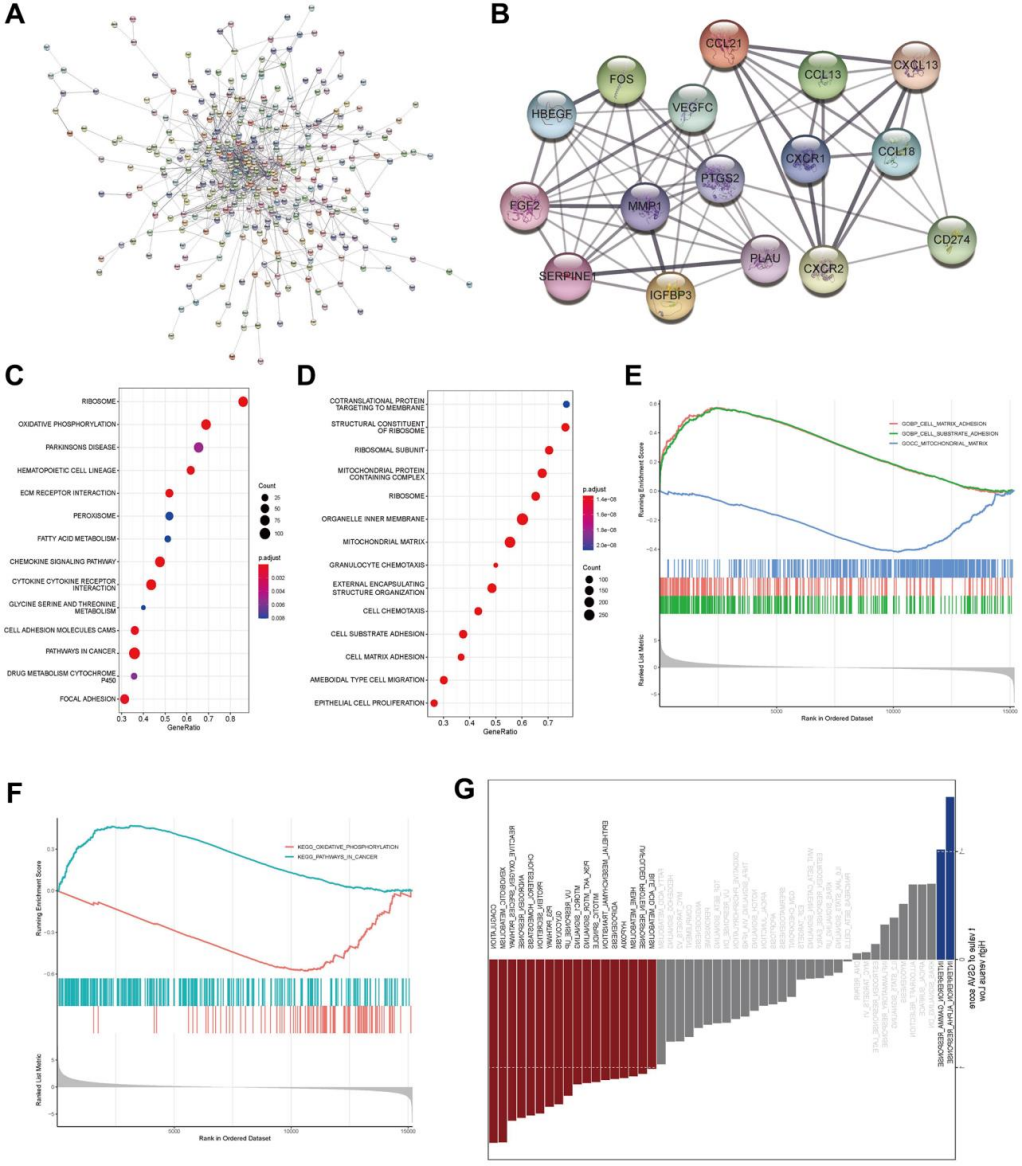
**Prediction of the functions of LncRNA AL161431.1 in CCA.** (**A**) The protein interaction network of differential mRNA in the high- and low- AL161431.1 expression groups was constructed using Cytoscape, and the confidence cutoff was set to 0.4. (**B**) The most relevant 16 key protein nodes were selected using the tool MCODE within Cytoscape. (**C**, **D**) GO functional annotation and KEGG enrichment of genes were analyzed using GSEA. (**E**, **F**) Further visualization was performed to examine whether the gene sets of these functions and signaling pathways obtained in GSEA analysis were enriched on the left or right side of the abscissa according to the degree of differential expression (LogFC) from high to low. (**G**) GSVA analysis in the hall-markers gene set showed that the high-AL161431.1 expression of group was mainly enriched in metabolic and tumor-related pathways.

To look into possible roles for AL161431.1 in CCA, we further looked at genes that were expressed differentially in the high- and low-expression groups of AL161431.1. After removing the genes that were not expressed in all tumor samples, the genes that remained were subjected to differential analysis using the edgeR algorithm in order to determine their logFC values. Based on the cut-off criterion of fold change >2.0 and *P* < 0.05, 442 genes were found to have differential expression. Using Cytoscape, the protein interaction network of the aforementioned differential genes was built as Figure 2A illustrates. The most relevant 16 key protein nodes were selected using the tool MCODE within Cytoscape, as shown in Figure 2B. Subsequently, the enrichment of genes was analyzed using GSEA. According to GO functional annotation, AL161431.1 was directly related to a variety of cell activities, including oxidative metabolism (mitochondrial matrix) and migration and invasion (cell substrate adhesion/cell matrix adhesion) (Figure 2C). These differential genes were also highly enriched in pathways linked to metabolism, such as oxidative phosphorylation pathways in cancer, and tumor metastasis, according to KEGG enrichment analysis (Figure 2D).

Further visualization was carried out in accordance with the significant functions and signaling pathways identified by the GSEA analysis in order to determine whether these gene sets were enriched on the left or right side of this abscissa in accordance with the degree of differential expression (LogFC) from high to low, results are displayed in Figure 2E, 2F. While mitochondrial matrix related to metabolism was enriched on the right side of the axis, suggesting that the high-risk metabolism-related function was inhibited, cell substrate adhesion and cell matrix adhesion associated with tumor metastasis and invasion were primarily enriched on the left side of the axis, indicating that this part of the function was activated in the high-risk group. Similarly, oxidative phosphorylation linked to oxidative metabolism was suppressed and pathways linked to tumors were activated at high expression group. Additionally, we downloaded the gene set of hallmarkers for GSVA analysis, and comparable findings indicated that the metabolic and tumor-related pathways were the primary areas of enrichment for the high-expression group (Figure 2G).

### Correlation between immune cell infiltration and AL161431.1 expression

**Figure 3:**
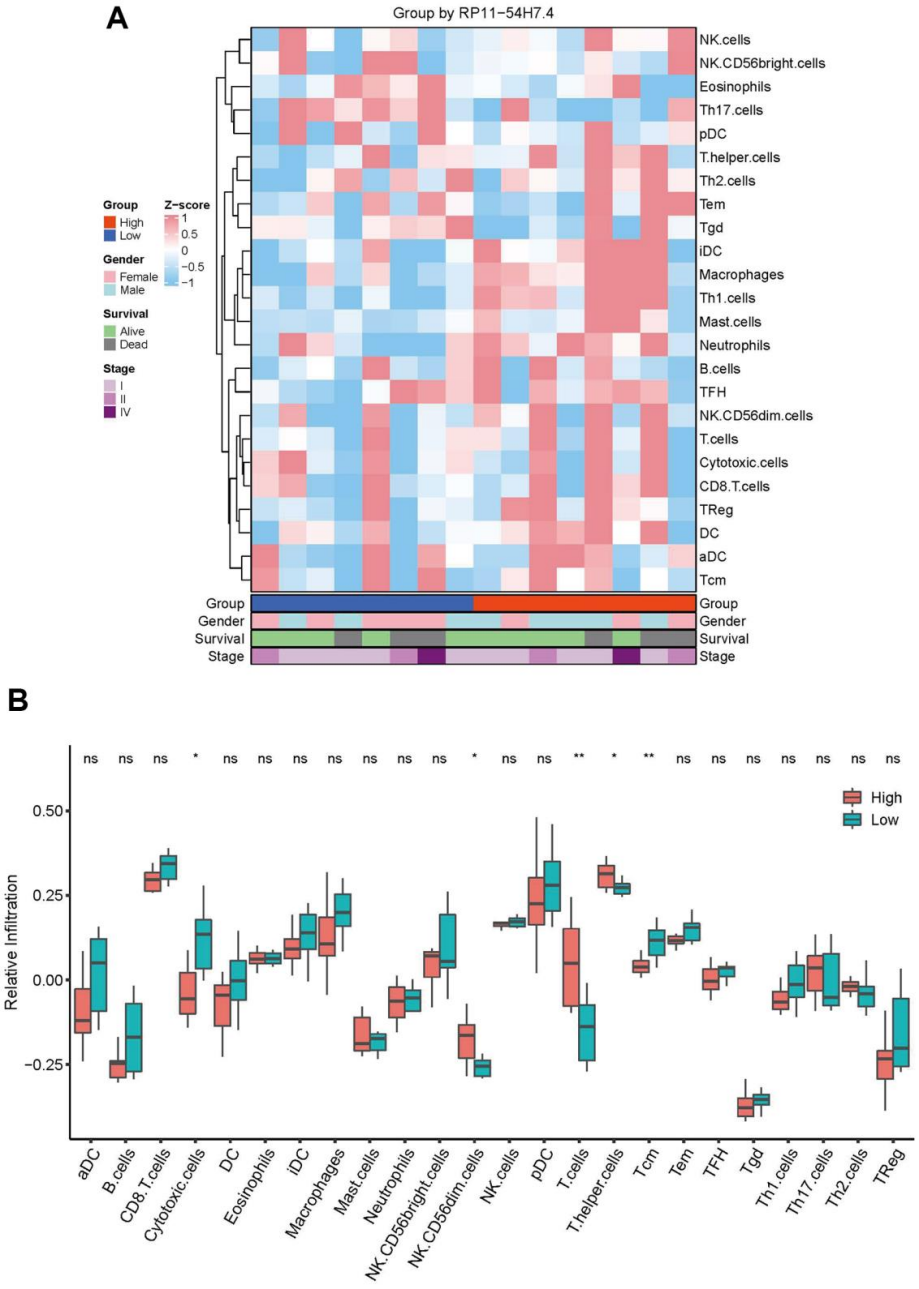
**Correlation between immune cell infiltration and AL161431.1 expression.** (**A**) Infiltration abundance of 24 immune cells in CCA samples was calculated by ssGSEA algorithm and transformed the sample-expression matrix into a sample-immune cell score matrix. (**B**) The abundance of 24 immune infiltrates in the high- AL161431.1 Expression and low- AL161431.1 Expression groups was compared by *t*-test. ^ns^*p* ≥ 0.05, ^*^p < 0.05, ^**^p < 0.01.

The association between immunity and AL161431.1 was next examined by contrasting the degrees of immune infiltration in the high- and low-risk groups. First, 24 immune cell gene sets were retrieved from a 2013 Immunity study titled “Synamotemporal Dynamics of Intra-tumoral Immune Cells Reveal the Immune Landscape in Human Cancer”. Following this, we extracted the mRNA-related gene expression levels from CCA samples, computed the infiltration abundance of these 24 immune cells in each sample using the ssGSEA algorithm, transformed the sample-expression matrix into a sample-immune cell score matrix, and then used the R language to visualize the immune cell enrichment in two groups after normalization using the z-score method. The results shown in Figure 3A suggest that the group at higher risk had a higher total degree of immune infiltration as compared to the group at lower risk.

To further support the disparities in immune cell infiltration between the two groups, the *T*-test was implemented to compare the quantity of 24 immune cells in the high-risk and low-risk groups. The results demonstrated that the low-risk group had higher infiltration abundances of Tcm and cytotoxic cells, the high-risk group had more expansive infiltration abundances of T cells, T helper cells, and NK CD56dim cells (Figure 3B). The results indicated a strong correlation between immune infiltration in CCA and high expression of AL161431.1.

### Effects of AL161431.1 on CCA cell proliferation, migration and invasion *in vitro*

**Figure 4:**
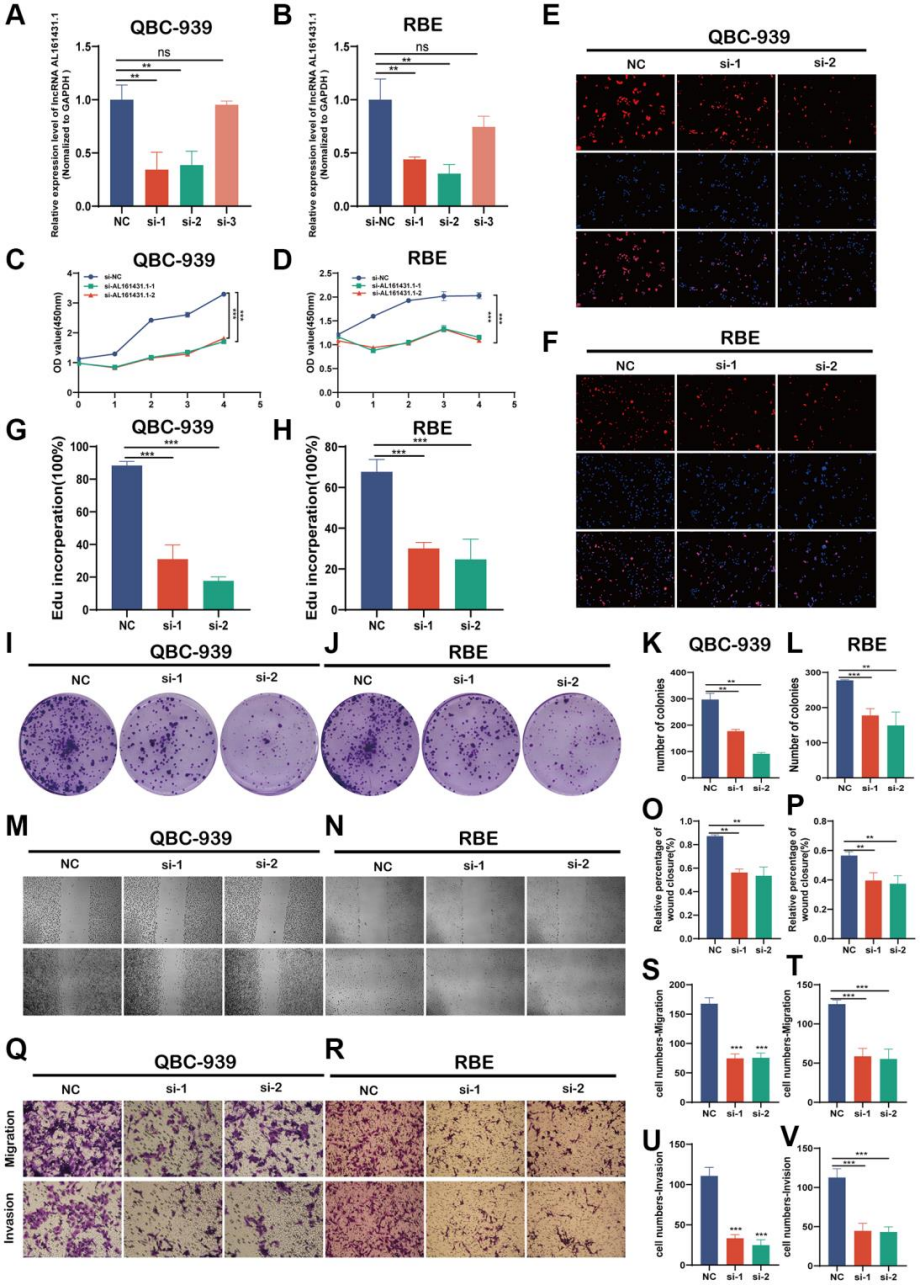
**AL161431.1 promotes CCA cell proliferation, migration and invasion *in vitro*.** (**A**, **B**) Expression levels of AL161431.1 in QBC-939 and RBE cells treated with three siRNA. (**C**–**L**). Cell proliferation detection of QBC-939 and RBE cells were measured by CCK-8, EDU assay and clone formation (magnification, x100, scale bar, 100 um). (**M**–**V**). Cell migratory and invasive capabilities were assessed by wound healing assays and transwell assays in QBC-939 and RBE cells transfected with NC, si- AL161431.1-1 and si- AL161431.1-2 (magnification, ×100, scale bar, 100 μm). ^ns^*p* ≥ 0.05, ^*^p < 0.05, ^**^p < 0.01, ^***^p < 0.001, ^****^p < 0.0001.

Three distinct siRNAs were created to reduce AL161431.1’s expression in CCA cell lines. The siRNA1 and siRNA2 effectively decreased the expression of siRNA in QBC-939 and RBE cells, as seen in Figure 4A, 4B. Growth curves by CCK8 assays showed that downregulation of AL161431.1 decreased the proliferation viability of QBC-939 and RBE cell (Figure 4C, 4D). Further investigation using EdU assays showed that AL161431.1 knockdown reduced the proportion of positive cells (Figure 4E–4H). Furthermore, the results of colony formation assays showed that downregulation of AL161431.1 considerably reduced the number of cell colonies in QBC-939 and RBE (Figure 4I–4L). These findings suggested that AL161431.1 may promote CCA cell proliferation. Also, trans-well assays and wound healing assays revealed a substantial reduction in the migratory and invasion capacities of CCA cells when deleted AL161431.1. (Figure 4M–4V).

### The mechanism of AL161431.1 in regulating cell functions

**Figure 5:**
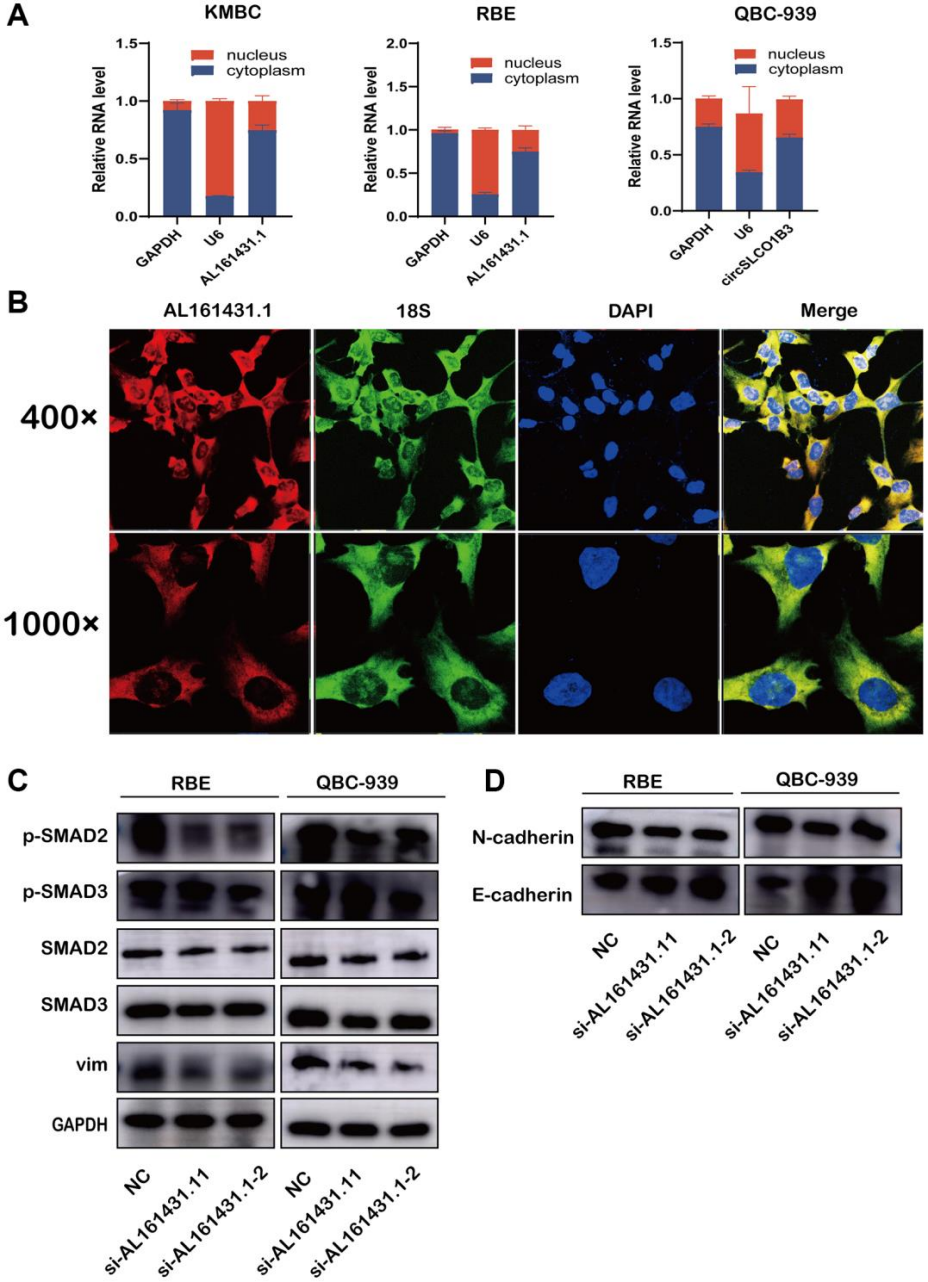
**AL161431.1 activates TGF-β/SMAD Pathway to promote EMT and its cellular location.** (**A**) qRT-PCR was used to measure the level of AL161431.1 in the nuclear and cytoplasmic of KMBC, QBC-939 and RBE cells. (**B**) FISH was performed to observe the cellular location of AL161431.1(red) and 18s(green) in RBE cells (magnification, ×400, scale bar, 20 μm and magnification, ×1000, scale bar, 10 μm). (**C**) Western blotting showed expression of different TGF-β/SMAD Pathway proteins and epithelial and mesenchymal after silencing AL161431.1 in QBC-939, and RBE cells. (**D**) Western blotting showed expression of epithelial and mesenchymal proteins after silencing AL161431.1 in QBC-939, and RBE cells.

There are several different ways that LNCRNAs carry out their activities. Typical approaches included participating in mRNA splicing, sponging miRNAs, and binding to proteins. According to certain reports, a lncRNA’s cellular localization may provide information about its molecular mechanism. Thus, we performed FISH and nuclear and cytoplasmic extraction assays. The findings demonstrated that AL161431.1 was primarily located in the cytoplasm, suggesting that AL161431.1 may interact with RNAs (such as miRNAs) or proteins that are primarily found in the cytoplasm (Figure 5A, 5B). KEGG enrichment analysis and GO functional annotation results indicated that AL161431.1 was associated with CCA metabolism and metastasis. To the best of our knowledge, the TGF-β/SMAD signaling pathway is involved in several different cellular activities, most notably the EMT process and the metabolic reprogramming of cancer-associated fibroblasts [9]. Thus, we wondered whether AL161431.1 could encourage CCA development and metastasis via turning on the TGF-β/SMAD signaling pathway. Using the western blot, we found the expression levels of SMAD2/SMAD3 were not changed abruptly, the expression levels of phosphorylated SMAD2/SMAD3 was lower by downregulating of AL161431.1 (Figure 5C). Additionally, suppression of AL161431.1 resulted in a substantial increase in E-cadherin expression and a decrease in N-cadherin and vimentin protein levels (Figure 5D).

## DISCUSSION

Recent data indicates that LNCRNAs are crucial for the development and spread of CCA tumors. [10–13]. The utilization of bioinformatic analyses was advantageous in discerning signaling pathways connected with LNCRNAs, which are essential for mechanistic investigations [14]. An overexpressed lncRNA called AL161431.1 was found in CCA tissues based on the TCGA-CHOL dataset, and it has a favorable link with the prognosis of CCA. Based on previous studies, AL161431.1 might potentially target miR-1252-5p and MAPK signaling in endometrial carcinoma, construct a rival endogenous RNA network in lung squamous cell carcinoma, and hasten the process of epithelial mesenchymal transition in pancreatic cancer. [8, 12, 13]. Our study’s KEGG enrichment analysis and GO functional annotation revealed that the enriched function and pathway were connected to tumor metastasis, metabolism, and other relevant topics. To test if this phenomenon was a coincidence, AL161431.1 was knocked down by 2 siRNAs in RBE and QBC-939 cell lines. The results of functional study demonstrated that AL161431.1 could promote the proliferation, metastasis and invasion of CCA cells. These results indicated that AL161431.1 was a cancer-promoting genes in CCA, which was consistent with previous findings.

Rich tumor microenvironment and desmoplasia are characteristic histological features of CCA. Numerous immune cell types, including natural killer (NK) cells, regulatory T lymphocytes (Tregs), tumor-associated neutrophils (TANs), tumor-associated macrophages (TAM), endothelial and lymphatic cells, and cancer-associated fibroblasts, have been linked to the development and metastases of CCA. [15]. Previous research indicated that T cells induce immunosuppressive responses through a variety of methods, including as the inactivation of cytotoxic, natural killer (NK), and antigen-presenting T cells in CCA by transforming growth factor-β1 (TGF-β1) and interleukin (IL)-10 [16]. It also reported that cancer-associated fibroblasts (CAFs), which were associated with worse clinical features and poor prognosis, could increase infiltration of NK CD56dim cells and T helper cells [17, 18]. Suppressive immune cells in the CCA TIME promote oncogenic signaling which leads to reduced cytotoxic T lymphocyte (CTL) and Tcm infiltration in tumour immune microenvironment of cholangiocarcinoma [19]. However, the function of these immune cells within the tumor microenvironment is highly complex, as they can promote or inhibit tumor progression at different times [19]. In our study, there was a correlation between the amounts of NK CD56dim cells, T cells, T helper cells, Cytotoxic Cells and Tcm and AL161431.1 expression in CCA patients, which indicated that AL161431.1 may be responsible for the maintenance of an immunosuppression status in CCA.

The biological behavior of tumor cells was greatly impacted by abnormally expressed LNCRNAs in CCA, and these LNCRNAs were implicated in the progression of tumors through a variety of mechanisms, such as competition for endogenous RNA (ceRNA) regulatory networks, association with functional proteins, activation of cancer-related signaling pathways, and epigenetic modification of gene expression. [10]. In our study, the results of FISH and Nuclear and cytoplasmic extraction assays showed that AL161431.1 is predominantly located in the cytoplasm, indicating molecular function of AL161431.1 may be performed in the cytoplasm. One of the primary signaling pathways that encourages the advancement of CCA is TGFβ. The TGFβ-mediated development of EMT in CCA cell lines is supported by several research. [20–22]. Here, we knocked down AL161431.1 using specific siRNAs, then, the western blot indicated that AL161431.1 could activate TGFβ pathway via the upregulation of phosphorylated SMAD2/SMAD3 to contribute to CCA metastasis. These results demonstrated that AL161431.1 may promote tumor progression via activating the TGFβ/SMAD signaling pathway in CCA.

### Limitations of the study

Our study provided a valuable prognostic predictor and treatment target in CCA, but there were still some limitations. Firstly, our study suggested that AL161431.1 was significantly upregulated in CCA. Thus, more research on AL161431.1 in the mouse model is required in order to stabilize AL161431.1 in CCA cell lines. the expression level of AL161431.1 in tumor tissue, and patient bodily fluids is also needed. Those allows AL161431.1 to potentially serve as a useful biomarker and treatment target in CCA. Thirdly, the mechanisms of regulating the development of CCA required further investigation. Furthermore, we gathered a cohort from the public databases of the TCGA; however, these data were not available for yellow or black cohorts and were mostly from Western nations with a high white population.

## CONCLUSIONS

We showed by bioinformatics analysis and mechanistic studies *in vitro* experiments that lncRNA AL161431.1 was involved in the pathogenesis of CCA by promoting proliferation, migration and invasion. LncRNA AL161431.1 could be a valuable prognostic predictor and treatment target in CCA.
